# Screening of Theaflavins That Inhibit Proliferation of Nasopharyngeal Carcinoma Cells Through Metabolomics Approaches

**DOI:** 10.1002/fsn3.70642

**Published:** 2025-07-31

**Authors:** Chunpeng Wan, Xiaomeng Hu, Puxiang Yang, Mingxi Li, Muhammad Farrukh Nisar, Zihao Zhou, Yudi Gan, Yi Cai, Zhonghua Liu

**Affiliations:** ^1^ Research Center of Tea and Tea Culture, College of Agronomy Jiangxi Agricultural University Nanchang China; ^2^ University and College Key Lab of Natural Product Chemistry and Application in Xinjiang, School of Chemistry and Environmental Sciences Yili Normal University Yining China; ^3^ Jiangxi Cash Crops Research Institute Nanchang China; ^4^ Ministry of Education and Jiangxi Key Laboratory of Crop Physiology, Ecology and Genetic Breeding Jiangxi Agricultural University Nanchang China; ^5^ Department of Physiology and Biochemistry Cholistan University of Veterinary and Animal Sciences Bahawalpur Pakistan; ^6^ Guangzhou Municipal and Guangdong Provincial KeyLaboratory of Molecular Target & Clinical Pharmacology, the NMPA and State Key Laboratory of Respiratory Disease, School of Pharmaceutical Sciences Guangzhou Medical University Guangzhou China; ^7^ Key Laboratory of Tea Science of Ministry of Education, National Research Center of Engineering Technology for Utilization of Functional Ingredients From Botanicals, College of Horticulture Hunan Agricultural University Changsha China

**Keywords:** invasion, migration, nasopharyngeal carcinoma, proliferation, theaflavin‐3,3′‐digallate

## Abstract

To study the inhibitory mechanism of theaflavins on nasopharyngeal carcinoma cells to define a solid Chinese herbal antitumor remedy through metabolomics approaches. Various concentrations of theaflavin‐3,3′‐digallate (TF3) were examined for their effects on proliferation, migration, invasion, and apoptosis of nasopharyngeal carcinoma (NPC) cells by CCK‐8, colony‐forming assay, wound healing assay, trans‐well migration assay, and Hoechst staining assay. Moreover, metabolomics analysis along with UHPLC‐Q‐TOF mass spectrometry and UHPLC‐Q‐Exactive Orbitrap mass spectroscopic analyses were also performed. (1) The CCK‐8 assay results highlighted the activity of CNE‐2 cells exposed to TF3 significantly declined in a concentration‐dependent way. (2) The colony formation assay elucidated TF3 inhibitory effects on the colony formation and proliferation of CNE‐2 cells and C666‐1 cells. The higher the concentration of TF3, the more obvious inhibition in cell proliferation was seen. (3) The scratch test results also confirmed the migration ability of CNE‐2 and C666‐1 cells following the treatment of TF3. By increasing the concentration of TF3, more significant inhibition in the migration and invasion of cancer cells has been observed. (4) Hoechst staining further confirmed that TF3 could induce apoptosis in both of these cancer cell lines in a dose‐dependent way. Metabolomics studies generated about 584 metabolic products of significant alterations in tea polyphenol‐treated NPC cells. Multivariate analyses (PCA, PLS‐DA, OPLS‐DA) help establish precise group separation and model reliability, while differential metabolites were mainly enriched in ABC transporter and amino acid biosynthesis pathways. These findings suggest that tea polyphenols modulate key metabolic networks associated with cancer cell proliferation. TF3 has significantly declined proliferation, migration, and NPC cell invasion.

## Graphical abstract

This study investigated the effects of theaflavin‐3,3′‐digallate (TF3) markedly suppresses the proliferation, migration, invasion of nasopharyngeal carcinoma cells while promoting apoptosis in a dose‐dependent manner. Metabolomic profiling identified 584 altered metabolites, revealing key disruptions in ABC transporter activity and amino acid biosynthesis. TF3 potentially inhibited NPCs growth through targeting metabolic disruption.

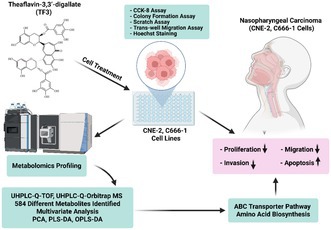

**Abbreviations**
CDDP: cisplatin
EBV: Epstein–Barr virus
EGCG: epigallocatechin gallate
HT: helical tomotherapy
IMRT: intensity modulated radiotherapy
NPC: nasopharyngeal carcinoma
OS: osteosarcoma
TF1: theaflavin
TF2a: theaflavin‐3‐gallate
TF2b: theaflavin‐3′‐gallate
TF3: theaflavin‐3,3′‐digallate
TFs: theaflavins

## 1 Introduction

Nasopharyngeal carcinoma (NPC) typically grows on the lateral wall in the nasopharynx, specifically in the posterior region, known as the pharyngeal recess (Al‐Ashqar and Khalil 2024; Jicman et al. 2022). The early symptoms of NPC often include intermittent bloody nasal discharge, with small amounts of bleeding (Jicman et al. 2022; Adham et al. 2020). As the tumor grows, it gradually narrows the nasal cavity, leading to nasal congestion (Adham et al. 2020). Initially, the congestion may be unilateral, but as the condition worsens, it can progress to bilateral nasal obstruction. In patients with tumors located in the pharyngeal recess, the tumor may compress the pharyngotympanic orifice (Varoquaux et al. 2013), resulting in symptoms such as tinnitus, a sensation of ear fullness, or hearing loss (Wolfe and Wilson 2007). NPC can also directly invade cranial nerves, causing symptoms such as migraines, numbness, and visual impairment (Chow et al. 2021).

Among numerous factors that may aid in the carcinogenesis of NPC, Epstein–Barr virus 4 (EBV‐4) is quite notable in the pathogenesis and progression of the disease (Wang et al. 2019; Su et al. 2023). EBV‐4 is primarily transmitted through saliva and proliferates within the epithelial cells of the oropharynx, posing a significant risk to the nasopharynx (Su et al. 2023). Moreover, the EBV‐4 genome integrates into the human host cell genome where it potentially influences carcinogenesis of NPC (Su et al. 2023). During NPC carcinogenesis, components of the EBV‐4 are released into the patients' bloodstream, triggering an immune response and leading to fluctuations in the levels of antibodies against EBV‐4 (Wong et al. 2021). Although EBV‐4 infection is widespread among populations worldwide, the distribution of NPC demonstrates regional variations (Alsavaf et al. 2025; Wu et al. 2025). This suggests that EBV‐4 is not the sole factor responsible for NPC, and other auxiliary factors may mediate the impact of EBV‐4 on its development. Additionally, the inhalation of tobacco smoke or other fumes increases the probability of developing NPC (Possenti et al. 2025; Lin et al. 2021). Research has shown that individuals who have smoked or been exposed to smoke have a comparatively higher (95%) risk of NPC than non‐smokers (WenQiong et al. 2013). The capacity of smoking to awaken dormant EBV‐4 infections might explain this. Studies also attempted to find that smoking is associated with elevated levels of anti‐EBV VCA IgA antibodies; more specifically, the percentage of positive serum antibodies increases in accordance with smoking intensity rather than time (Lin et al. 2021). Smoking also increases the incidence of non‐proliferative cervical cancer via anti‐EBV VCA IgA rather than anti‐EBV EA‐EBNA IgA (Hsu et al. 2020). Here we find evidence that smoking modifies the host's response to EBV infection, which may explain why it raises the risk of NPC (Hsu et al. 2020). Other factors, such as diet, occupation, and exposure to harmful gases, also contribute to the risk of developing NPC (Chen, Chang, et al. 2021). NPC tends to metastasize early, with a high rate of metastasis to cervical lymph nodes. Due to the location of NPC cells along the rich network of lymphatic vessels, separated only by the nasal mucosa, lymphatic drainage can extend across the entire neck, leading to an early occurrence and high rate of metastasis. The likelihood of distant metastasis in NPC increases with the enlargement and increased number of cervical lymph nodes (Lin et al. 2023). NPC is common among indigenous peoples in China, Southeast Asia, Arctic zones, Northern African countries, and the Middle East, among other places. Intensity‐modulated radiation therapy (IMRT) is a technique that uses various physical methods to adaptively adjust the shape and size of the planned target volume, altering the intensity of different radiation beams to deliver lethal doses to the target area while keeping the dose to normal areas below the tolerance level (Li et al. 2015). Helical tomotherapy (HT) is an emerging IMRT technique that combines CT scanning, dose calculation, and helical irradiation treatment (Lingling et al. 2023). It can focus on tumor irradiation in a 360° manner, selectively irradiating the target area with uniform intensity while avoiding normal tissues and organs, thus protecting them from the harmful effects of radiation therapy. HT also offers image‐guided treatment, allowing for precise radiation therapy by performing CT scans before treatment to confirm the three‐dimensional spatial shape and size of the treatment target area. It can also calculate the intensity dose of tumor irradiation after each treatment, adjusting subsequent treatment intensity levels to avoid causing further harm to the patient. This technology provides potential for improving target configuration and preserving critical structures. Additionally, due to the irregular shape of tumor tissues, their volumes align well with the goals of HT, making it particularly suitable for NPC (Liangpeng and Shanbin 2018; Yang et al. 2019). Despite NPC being sensitive to radiation therapy, for patients in the middle and late stages, radiation therapy alone can lead to metastasis or recurrence. Therefore, the current effective treatment approach for locally advanced NPC involves combining radiation therapy with cisplatin and fluorouracil, or with paclitaxel and nedaplatin chemotherapy. Research has shown that berberine treatment significantly affects the growth, proliferation, and migration ability of NPC cells. It can alter the expression patterns of NPC cells, inhibit oncogenic signaling pathways such as AKT/mTOR, and induce autophagic cell death (Rong 2021). Cyclooxygenase‐2 (COX‐2) is a potential oncogene that is closely related to the occurrence and progression of tumors. Triptolide may exert anti‐NPC cell activity through various mechanisms, including COX‐1 and COX‐2 inhibition, protein stabilization, and lysosome stabilization (Shipton et al. 2017).

Plant polyphenols (plant tannins) are a class of important natural products widely present in plants, such as dark berry (Jerome et al. 2022), apples, black tea, etc. Theaflavins (TFs) are a group of secondary polyphenolics derived from the metabolism of flavan‐3‐ols. TFs are abundantly present in black tea, which accounts for 2%–6% by dry weight (Sen and Bera 2013). Studies show that tumor factors have promoted cellular death along with a decline in cancer cell proliferation, migration, and survival (Jian et al. 2022). Due to the presence of a unique benzopyran‐4‐one structure, TFs possess direct or indirect antibacterial effects (Chunhua et al. 2017). Moreover, TFs can regulate underlying cellular mechanisms in the majority of cancer cells, and hence exert strong anti‐tumor effects (Huifan et al. 2021). Cytoprotective factors such as B‐cell lymphoma 2 (Bcl‐2) are downregulated following TFs exposure, while an increase in the expression levels of cell apoptotic factors such as poly(ADP‐ribose) polymerase (PARP), and caspases‐3, ‐7, ‐8, and ‐9 has been reported. The tumor suppressor p53 is highly upregulated in response to TFs exposure, whereas Akt, mTOR, PI3K, and c‐Myc levels are decreased (O'Neill et al. 2021).

Drug resistance is one of the major challenges in treating breast cancer, particularly estrogen receptor‐positive breast cancers against tamoxifen. TFs have the potential to suppress aromatase, which in turn lowers the resistance in breast cancer cells against tamoxifen. In addition to inhibiting angiogenesis, flavonols can block the androgen receptor in prostate cancer cells, which in turn reduces the amount of vascular endothelial growth factor (VEFG) expression in tumor lysates (Siddiqui et al. 2006). Both TFs and TF3 can suppress cell growth and extracellular signal transduction (Koňariková et al. 2015). Tumor necrosis factor α (TNFα) can upregulate Bax expression, which counteracts the cytoprotective role of Bcl‐2 and regulates the cell cycle, ultimately leading to cell apoptosis (Halder et al. 2006). Significant cellular death in breast epithelial carcinoma (Lakshmishri et al. 2008) and leukemia cells (Schuck et al. 2008) has been reported following TFs treatment, which induces cell shrinkage, vesiculation, and mitochondrial fragmentation. Studies found that the cytotoxicity of the tea mold mixture grows with time and that CAL27 cells are more susceptible than immortalized GT1 leukocytes. In human oral cavity normal HGF‐2 cells, TFs mitigate cytotoxicity by lowering the production of H_2_O_2_ in the presence of catalase and CoCl_2_, which later catalytically decompose H_2_O_2_. TFs potentially inhibit NF‐κB and interfere with the ROS‐mediated p53 signaling cascades in order to prevent cancer cell migration (Adhikary et al. 2009). Cervical cancer HeLa cells treated with TF1 showed an increase in upregulation in Bax expression, while Bcl‐2 and matrix metalloproteinases (MMPs) are downregulated. In addition, excessive ROS burst with minimal levels of GSH content has been seen. Moreover, TF9‐treated HeLa cells released more cytochrome C, activated caspase‐3, activated caspase‐1, and cleaved PARP. The inactivation and suppression of NF‐κB nuclear translocation occurred as a result of a decline in phosphorylated IκBα. HeLa cells treated with tea flavonols had lower levels of cyclin D1, phosphorylated Akt (Ser473), and Cox‐2 expression (Madhulika et al. 2011). Tea flavonols also enhanced cisplatin‐induced apoptosis and increased cisplatin sensitivity in HeLa cells, as TFs can modulate the expression of NF‐κB signaling pathway‐related proteins (Liping et al. 2018). TF2 declines transcriptional activation of COX‐2, providing a potential therapeutic approach for cancer and other inflammation‐related diseases (Alexander et al. 2011).

Ascorbic acid in combination with EGCG or TF3 induces apoptosis in SPC‐A‐1 and ECA‐109 cells by activating caspase‐3 and ‐9 expression (Gao et al. 2013). One possible mechanism by which tea flavonols protect against oral cancer and dental caries is mainly by regulating the hydrolysis of tea flavanol gallates (Mao‐Jung et al. 2004). Cellular apoptotic body formation and chromatin condensation were shown to be elevated in human diffuse large B‐cell lymphoma U3 cells treated with TFs, which are obvious markers of accelerated apoptosis. As a result, flavonols found in tea may have therapeutic potential for leukemia treatment. TF1, TF2a, TF2b, or TF3 treatment dramatically reduced the growth and promoted apoptotic cell death in OVCAR‐3 and A2780/CP70 ovarian cancer cells (Saeki et al. 1999), and inhibited the anti‐apoptotic protein Bcl‐xL. Tea flavonol administration raised the levels of cleaved caspases (caspase‐3, ‐7, ‐8, and ‐9), pro‐apoptotic Bax protein, FAD, and death receptor 5 (DR5). A stronger pro‐apoptotic response was induced by TF2a, TF3b, and TF1 than by TF2 (Ying et al. 2016).

Anti‐herpes simplex virus (HSV) activity of tea flavonols is ranked as follows: TF3 > TF2 > TF1 (de Oliveira et al. 2015). TF3 was found to have no toxicity while preventing the production of HSV‐1 viral plaques more efficiently than TF1 or TF2. The efficiency of tea flavonols against HSV‐1 infection in Vero and A549 cells was confirmed by reduced GFP expression in vitro when exposed to an elevated concentration of TF1, TF2, or TF3. TF3 can prevent the binding of the virus to Vero and A549 cells and inhibit its ability to complete the lytic cycle, suggesting that it directly acts on HSV‐1 viral particles to hinder their binding or penetration into cells (de Oliveira et al. 2015). However, once the virus has penetrated the cells, TF3 does not exhibit any inhibitory effects.

Like EGCG, TF3 boosts oxidative stress to pose strong anti‐proliferating effects and selectively kills cancer cells without any harm to normal cells (Kundu et al. 2005; Babich et al. 2006). In order to increase the sensitivity of ovarian cancer to cisplatin (cDDP), EGCG regulates cisplatin influx transporter CTR1. Due to their synergistic induction of cell cycle arrest, EGCG and cDDP may have multi‐fold effects against cholangiocarcinoma cells. Produced from EGCG, TF3 potentially alleviates cDDP adverse effects and overall tumor resistance was declined. Compared with ovarian cancer cells, TF3 demonstrates lower cytotoxicity towards normal OSE‐364 cells. The combination of TF3 and cDDP is more efficient than either drug alone in killing cisplatin‐resistant ovarian cancer cells. Another potential utilization of TF3 is as an adjuvant therapy for advanced ovarian cancers (Pan et al. 2018). Ovarian cancer cells undergo apoptosis and G1/S cell cycle arrest as a result of its synergistic effects on cDDP. TF3 induces oxidative stress that halts cell growth by triggering cell cycle arrest and limiting survival of human osteosarcoma cells along with altered cellular iron metabolism. By triggering excessive ROS generation and activation of the MAPK pathway, TF3 suppresses osteosarcoma growth in heterologous tumors (Tao et al. 2022).

Currently, metabolomics‐based available literature highlighted that tea polyphenolic compounds have a significant potential to distort cellular metabolic routes in tumor cells, which is deemed a crucial step in actively proliferating cancer cells (Trisha et al. 2022). Cancer cells may undergo metabolic reprogramming in order to maintain quick proliferation and survivorship under multiple microenvironmental stressors, for example, Warburg effect, and high rates of glycolytic activity to cope with increased energy demands even under low O_2_ supplies (Schiliro and Firestein 2021). Tea polyphenols have extensively been reported to suppress this condition and downregulate the production of intermediatory compounds of the glycolysis pathway while boosting oxidative phosphorylation. Moreover, tea polyphenols regulate metabolism, lipogenesis, cross‐talks of nucleotide biosynthetic pathways, and deamination and transamination of amino acids in actively dividing cancer cells (Chen, Cheng, et al. 2021). These metabolic involvements of polyphenols not only suppress tumor growth but also help sensitize cancer cells against chemotherapeutic agents and radiotherapies.

Furthermore, tea polyphenol metabolomic studies confirmed a modulation in tumor microenvironmental niches by regulating immune cell metabolism and inflammation‐associated cell signaling (Xie et al. 2012). Various cellular phase‐II enzymes such as glutathione (GSH) and heme oxygenase‐1 (HO‐1) quickly respond to restore cellular redox balance, which was previously disorganized due to metabolism‐associated ROS bursts. Moreover, tea polyphenols not only restore cellular redox balance, but also shield against DNA damage (Truong and Jeong 2021). Exposure to polyphenols is linked with lower levels of metabolic products actively taking part in angiogenesis and metastasis, hence confirming their potent cell regulatory roles (Truong and Jeong 2021). Metabolomic mapping unveils the systemic effects of tea polyphenols, specifically their impact on host metabolism, gut microbiota‐mediated metabolic products, and serum biomarkers, making them promising agents for sustainable anticancer therapeutic strategies.

Tea TFs comprised of a group of polyphenolic compounds predominantly found in black tea, showed significant anticancer potential against NPC cells following different signaling pathways (Trisha et al. 2022). EGCG has been reported to pose significant chemopreventive and therapeutic properties in numerous cancers (Sehgal et al. 2023). Metabolomic studies have played a pivotal role in elucidating the biochemical pathways influenced by tea polyphenols in tumor cells. The application of the latest metabolomics techniques, alterations in key metabolic intermediates and signaling pathways following TFs exposure may transform the metabolic programming of cancer cells that may hinder their proliferation and survival. In addition, metabolomic profiling enables the identification of specific biomarkers and metabolic signatures associated with tea polyphenol intervention across different cancer types. There is substantial evidence that tea flavonol TF3 can effectively suppress tumor cell motility, invasion, and proliferation as well as induce cell cycle arrest along with the promotion of apoptosis. Several mechanisms have been suggested through mechanistic studies including ERK and JNK pathways, as well as the modification of MMPs expression patterns. In order to define a solid Chinese herbal remedy for cancer treatment with no or fewer side effects and enhancing immunity, the present study attempted to find how TF3 affects the migration, proliferation, and invasiveness of human NPC cells.

## 2 Materials and Methods

### 2.1 Culturing of Cells

Human NPC CNE‐2 and C666‐1 cells were obtained from the Shanghai Institute of Biological Sciences and preserved in our laboratory. The culture medium was prepared as follows: 5 mL of FBS (Thermo Fisher Scientific, USA), 0.5 mL of a mixture of 50 U/mL penicillin and 50 U/mL penicillin–streptomycin (Thermo Fisher Scientific, USA), and 44.5 mL of serum‐free DMEM medium (Thermo Fisher Scientific, USA) were sequentially added to a 50 mL centrifuge tube to obtain 50 mL of complete culture medium. The cells were cultured at 37°C under a humidified incubator with 5% CO_2_. Every 24 h, the cells were rinsed by using a sterilized PBS (Thermo Fisher Scientific, USA), and the medium was replaced with fresh DMEM containing antibiotics (1%) and FBS (10%). Every 3 days, or when the cell growth exceeded 80% confluency, the cells were passaged using pancreatin (Thermo Fisher Scientific, USA) and divided into 2–3 dishes for separate culture or cryopreservation. All experimental cells used in the following experiments were cultured until they reached 80% confluency.

### 2.2 Preparation of Theaflavins

The four main types of theaflavins (P & S Biotechnology Co. Ltd.), including teaflavin‐3,3′‐digallate (TF3), teaflavin (TF1), teaflavin‐3‐gallate (TF2a), and teaflavin‐3′‐gallate (TF2b), were dissolved in different amounts of DMSO (MP Biomedicals) and prepared into solutions of the same concentration gradient (12.5, 25.0, and 50.0 μM).

### 2.3 CCK‐8 Assay

CNE‐2 cells (5000/well) were planted in a 96‐well plate and were maintained in DMEM with FBS (10%) and antibiotics (1%) and kept under 37°C in a CO_2_ equipped cell culture incubator. After about 24 h, the antibiotic‐free DMEM medium with 1% FBS was swapped out for the old one. The drug group received theaflavins (12.5, 25.0, and 50.0 μM), specifically TF3, TF1, TF2a, and TF2b, whereas the non‐treated group received an equal volume of DMSO. Six replicates were conducted for each concentration of each medication in a different plate. Following the incubation period, the previous medium was replaced with 10% CCK‐8 solution (100 μL) (DOJINDO Laboratories, Japan) in serum‐free DMEM, which was added to every well and allowed for an hour in the incubator. Following incubation, absorbances were measured in an ELISA reader at 450 nm. As an indicator of the cell survival rate throughout a range of treatment durations and doses, absorbance was used to compute cell viability.

### 2.4 Colony Formation Assay

A density of 1000 cells per well was used to seed CNE‐2 and C666‐1 cells onto 6‐well plates. In DMEM medium with 1% antibiotics and 10% FBS, the cells were grown until they attached fully. Following full adhesion, several amounts of TF3 (12.5, 25.0, and 50.0 μM) were introduced to every well, whereas the negative control group received an identical volume of DMSO solvent. Triplicates of each concentration were examined. During the 2 weeks of cell culture, the medium was replaced every 2 days while the medication concentration remained the same. In order to prepare the cells for staining, 1.0 mL crystal violet solution (0.5%) made in methanol (25%) was added to each well following two washes with PBS. The previous medium was then discarded. In a single hour at room temperature, the plate took on a stain. Next, we gently washed off any leftover stain with water after removing the crystal violet staining solution. Following an air drying period, the plate was imaged utilizing a gel imaging equipment. We tallied all the cell colonies that included fifty or more cells. A relative colony formation rate (%) was found by dividing the number of cell colonies in the drug group (> 50 cells) by the number of cell colonies in the non‐treated group (> 50 cells) × 100%.

### 2.5 Scratch Assay

The back of a 6‐well plate was marked with three equally spaced horizontal lines using a marker pen. The 6‐well plate was seeded with 3 × 10^4^ cells per well of CNE‐2 and C666‐1 cells. The 2 mL of DMEM media that included 1% antibiotics and 10% FBS was added to each well. Three wells were set up in parallel for each group. Overnight, the plate was placed in the cell incubator to allow the cells to form a monolayer. After the cells completely covered the bottom of the plate, three scratches were made on each well using the tip of a 200 μL pipette with equal force perpendicular to the horizontal lines on the back of the plate. The medium was aspirated, and 1 mL of PBS solution was gently poured into each well to wash away the remaining cell debris from the scratches. The PBS solution was aspirated, and the serum‐free DMEM medium was replaced. The wells were photographed at the same position under a microscope (0 h), followed by a series of TF3 concentrations (12.5, 25.0, and 50.0 μM) exposure to each well, with the same quantity of DMSO used as the negative control group. The plate was placed in the cell incubator for further incubation. After 24 h, the wells were photographed at the same position under a microscope to observe the healing ability of the cells in the scratches.

### 2.6 Trans‐Well Invasion Assay

The concentration of Matrigel (Corning, USA) was reduced to 50 mg/L by diluting (1:8) it DMEM without serum. In the upper chambers of Trans‐well insert was filled with 45 μL of the diluted Matrigel solution, and then allowed it to incubate at 37°C in a 5% CO_2_ cell incubator for 2 h until it was solidified. CNE‐2 and C666‐1 cells in logarithmic growth phase were collected and diluted in 0% DMEM to a volume having 5 × 10^4^ cells/mL. The cell suspension in the drug group was supplemented with TF3 (12.5, 25.0, and 50.0 μM), whereas same volume of DMSO was taken as negative control group. In four independent experiments for one concentration level 200 μL cell suspension was added in the upper chamber of the Trans‐well insert that included varying amounts of the drug solution or DMSO. The lower chamber of the Trans‐well insert was supplemented with 600 μL of DMEM supplemented with antibiotics (1%) and FBS (10%). Set aside for 24 h in an incubator. When the incubation time is up, take out the culture medium and give everything a good washing with PBS. About 1000 μL of paraformaldehyde (4%) was added in each well of 24‐well plate. (Guangdong Guanghua Technology Co. Ltd.), gently placing it in the Trans‐well chamber at room temperature for 30 min. Remove the excess 4% paraformaldehyde, wash twice with PBS, and remove any remaining liquid. 1000 μL crystal violet (0.1%) solution (Dalian Meilun Biotechnology Co. Ltd.) was added to each well of 24‐well plate, gently placing it in the Trans‐well chamber and allowing it to stain at room temperature in dark for about an hour. Later on, collected the crystal violet solution, rinse off any remaining residues gently with water, and allow it to air dry before imaging using an inverted microscope (10×).

### 2.7 Hoechst Staining Experiment

C666‐1 and CNE‐2 cells were placed in a 12‐well plate having about 9 × 10^4^ cells in each well, along with 1000 μL DMEM supplemented with bispecific antibody (1%) and FBS (10%), and allowed to incubate for another 24 h. After 24 h, the supplemented medium was replaced with fresh DMEM with no serum, and TF3 (12.5, 25.0, 50.0 μM) was added to different designated wells. A similar quantity of DMSO was added in negative controls. Each TF3 concentration was replicated three times, and the plate was again placed for an additional 24 h for incubation. Later on, DMEM was aspirated, 500 μL of fixing solution was added and let it be for 10 min. Following the removal of the fixation solution, cells were gently washed twice using PBS before adding 500 μL of Hoechst 33258 staining solution (Shanghai Biyuntian Biotechnology Co. Ltd.) at ambient temperature for 5 min. The Hoechst solution was then removed, and cells were washed twice using PBS before the addition of 50 μL of anti‐fluorescence quenching mounting media. The plate was examined and imaged using a fluorescence inverted microscope (20×) in a light‐restricted setting.

### 2.8 Metabolomics Analysis

#### 2.8.1 Cultured Cells

The culture media was taken from the CNE‐2 cells, which had been cultured at around 10^7^ cells per sample, using a pipette. The next step was rinsing the cells with PBS at 37°C and then eliminating the PBS. To facilitate protein removal and metabolite extraction, cold methanol/acetonitrile (1:1, v/v, 800 μL) was employed. Now the mixtures were collected into new tubes, spun at 16434 g for about 15–20 min, and then the supernatants were harvested. Using a vacuum centrifuge, the liquid was evaporated to get the solids behind, which were again mixed with 100 μL of equal volumes of acetonitrile/water (v/v) for LC–MS analysis. This mixture was spun for about 15–20 min under 14,000 *g* at 4°C, and the resultant supernatant was injected. The instrument's analytical consistency and reliability were assessed by amalgamating 10 μL of every sample to formulate quality control (QC) samples, thereafter analyzed alongside the remaining samples. Every fifth sample was analyzed following the systematic insertion of quality control samples.

#### 2.8.2 UHPLC‐Q‐TOF Mass Spectrometry Analyses

The samples were analyzed commercially by Applied Protein Technology (Shanghai, China) through a quadrupole time‐of‐flight mass spectrometer (AB Sciex TripleTOF 6600) equipped with an ultra‐high‐performance liquid chromatography system (Agilent Technologies 1290 Infinity LC). Sample separation was achieved through hydrophilic interaction liquid chromatography (HILIC) using an ACQUIY UPLC BEH Amide column (1.7 μm, 2.1 mm × 100 mm) from Waters, Ireland. For positive and negative electrospray ionization (ESI) modes, the mobile phase consisted of ammoniumcetate (25 mM) and ammoniumhydroxide (25 mM) solutions prepared in H_2_O, with acetonitrile as solvent B. The gradient program started with 95% solvent B for 30 s, followed by a linear reduction to 65% over 6.5 min. It was then decreased to 40% within 1 min and held at this level for an additional minute. Subsequently, the solvent B concentration was increased back to 95% in a few seconds, followed by a 3‐min re‐equilibration period. The ESI source parameters were set as follows: Ion Source Gas1 (Gas1) and Ion Source Gas2 (Gas2) were both set to 60 V, the curtain gas (CUR) to 30 V, and the source temperature to 600°C. The ion spray voltage floating (ISVF) was adjusted to ±5500 V. The mass spectrometer operated in MS‐only mode, covering an *m*/*z* range of 60–1000 Da, with an accumulation time of 0.20 s per spectrum for TOF MS scans. Automated MS/MS acquisition was configured to capture data within an *m*/*z* range of 25–1000 Da, with a product ion scan accumulation time of 0.05 s per spectrum. Information‐dependent acquisition (IDA) was employed in high sensitivity mode to obtain product ion scans. Key parameters included a collision energy (CE) of 35 V with a standard deviation of 15 eV, a declustering potential (DP) of ±60 V, and the exclusion of isotopes within a 4 Da range. Additionally, up to 10 candidate ions were monitored per cycle.

#### 2.8.3 UHPLC‐Q‐Exactive Orbitrap Mass Spectroscopic Analyses

This analysis was performed using an Orbitrap mass spectrometer integrated with a UV‐HPLC system (Vanquish UHPLC, Thermo) (Shanghai Applied Protein Technology Co. Ltd.). Sample separation was done via hydrophilic interaction liquid chromatography (HILIC) using an ACQUITY UPLC BEH Amide column (1.7 μm, 2.1 mm × 100 mm) from Waters, Ireland. For both positive and negative electrospray ionization (ESI) modes, the mobile phase consisted of an ammonium‐acetate (25 mM) solution and an ammonium‐hydroxide (25 mM) solution prepared in distilled H_2_O, with acetonitrile serving as solvent‐B. The gradient elution program was adjusted as follows: an initial hold at 98% solvent B for 1.5 min, followed by a linear reduction to 2% over 10.5 min. This composition was maintained for 2 min before rapidly increasing back to 98% within 0.1 min, followed by a 3‐min re‐equilibration phase. The ESI source parameters were configured as follows: Ion Source Gas1 (Gas1) and Ion Source Gas2 (Gas2) were both set to 60 V, the curtain gas (CUR) to 30 V, and the source temperature to 600°C. The ion spray voltage floating (ISVF) was maintained at ±5500 V.

For MS‐only acquisition, the instrument was set to scan an *m*/*z* range of 80–1200 Da, with a resolution of 60,000 and an accumulation time of 100 milliseconds. During automated MS/MS acquisition, the parameters included an *m*/*z* range of 70–1200 Da, a resolution of 30,000, an accumulation time of 50 milliseconds, and an exclusion duration of 4 s.

#### 2.8.4 Data Processing

The raw data obtained from mass spectrometry (MS) was initially converted into MzXML files using ProteoWizard MS Convert, and followed its import into the open‐source XCMS software for further processing. The following parameters were applied for peak selection: centWave *m*/*z* = 10 ppm, peak width = *c*(10, 60), and prefilter = *c*(10, 100). For peak grouping, the settings included bw = 5, mzwid = 0.025, and minfrac = 0.5. Isotope and adduct annotation was performed using CAMERA (Collection of Algorithms for Metabolite Profile Annotation). To maintain data quality, only ion features with more than 50% nonzero measurement values in at least one group were retained. Metabolite identification was conducted by matching the accuracy of *m*/*z* values (< 10 ppm) and MS/MS spectra against an in‐house database built using authentic reference standards.

### 2.9 Statistical Analysis

The statistical analysis was done using GraphPad Prism (version 8.0), and results are given as mean ± standard deviation (SD). Variance was assessed using one‐way ANOVA, with a significance level *α* = 0.05, where a *p* value of < 0.05 was considered statistically significant. Multivariate data analyses, including Pareto‐scaled principal component analysis (PCA) and orthogonal partial least‐squares discriminant analysis (OPLS‐DA), were performed on the processed data after sum‐normalization using the R package *ropls*. The robustness of the model was evaluated through response permutation testing and seven‐fold cross‐validation. In the OPLS‐DA model, the variable importance in projection (VIP) value was calculated to determine the contribution of each variable to the classification. Significant differences between two independent sample groups were assessed using Student's *t*‐test. Metabolites with a VIP value > 1 and a *p* value < 0.05 were identified as significantly altered. Additionally, Pearson's correlation analysis was employed to assess the strength of relationships between variables.

## 3 Result

### 3.1 TFs Effects on CNE‐2 Cells Proliferation

**FIGURE 1:**
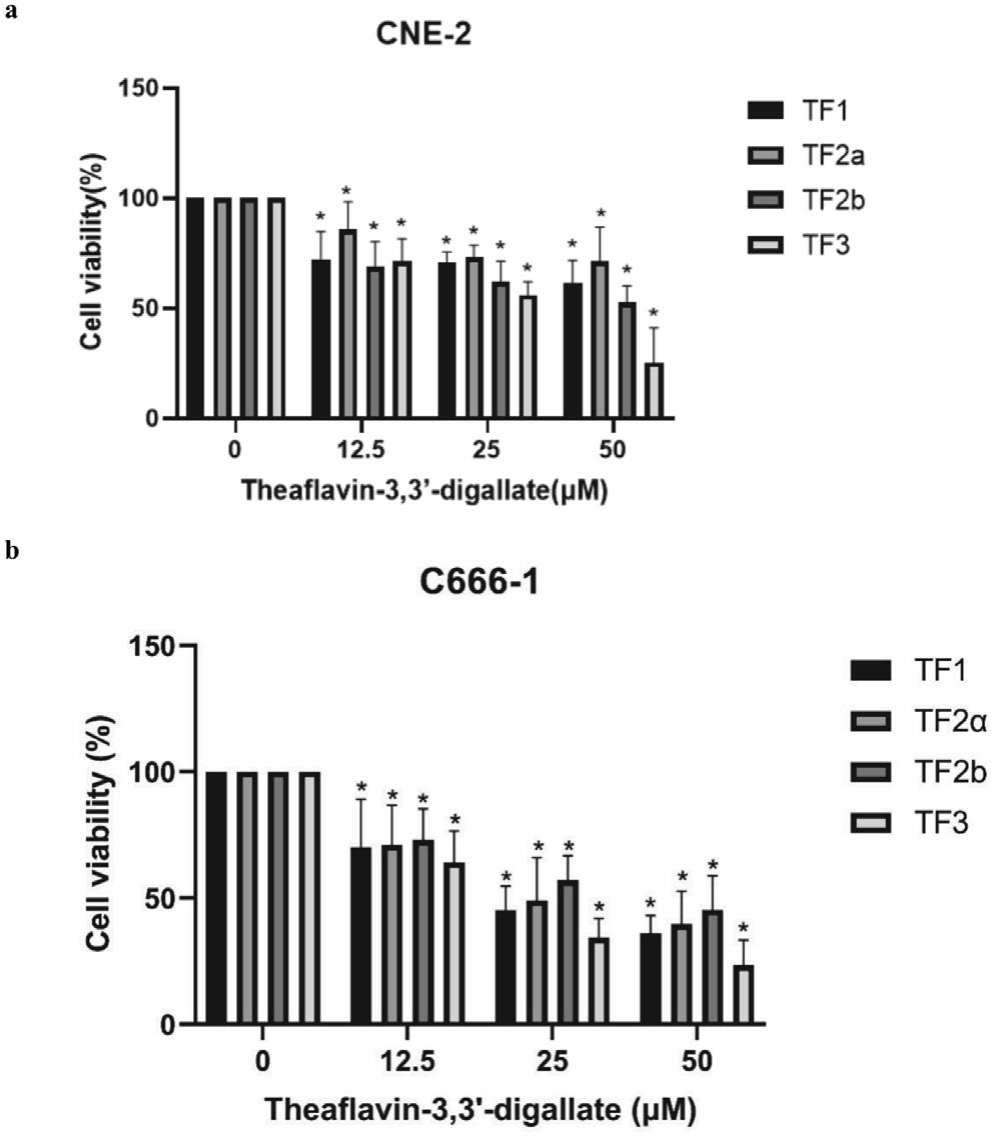
The effect of variable concentrations of TFs on the viability of (A) CNE‐2 cells and (B) C666‐1 cells (*p < 0.05, *n*3).

After treatment with four types of theaflavins, the viability of CNE‐2 cells decreased generally, with a more pronounced decrease observed after TF3 treatment. The cell viability of CNE‐2 and C666‐1 cells was assessed using the CCK‐8 assay (Figure 1A,B). The absorbance of both cell lines exposed to variable TF concentrations (0, 12.5, 25.0, and 50.0 μM) was measured. The results revealed that all four drugs exhibited certain inhibitory effects on human NPC CNE‐2 and C666‐1 cells, showing a dose‐dependent relationship. Among them, the TF3 group exhibited a more stable cell survival rate compared to TF1, TF2a, and TF2b, with lower cell survival rates observed in the 25 and 50 μM groups compared to the other three drugs, reaching a peak inhibition at 50 μM. These results indicate that TF3 exhibits a more pronounced inhibitory effect on CNE‐2 cells compared to TF1, TF2a, and TF2b, suggesting TF3 as the primary research drug for the subsequent cell experiments (Figure 1A). Interestingly, a pattern quite similar to that of CNE‐2 cells was shown by C666‐1 cells following different concentrations (0, 12.5, 25.0, and 50.0 μM) of TFs (Figure 1B).

### 3.2 Effect on Inhibition of Clonogenic Proliferation

**FIGURE 2:**
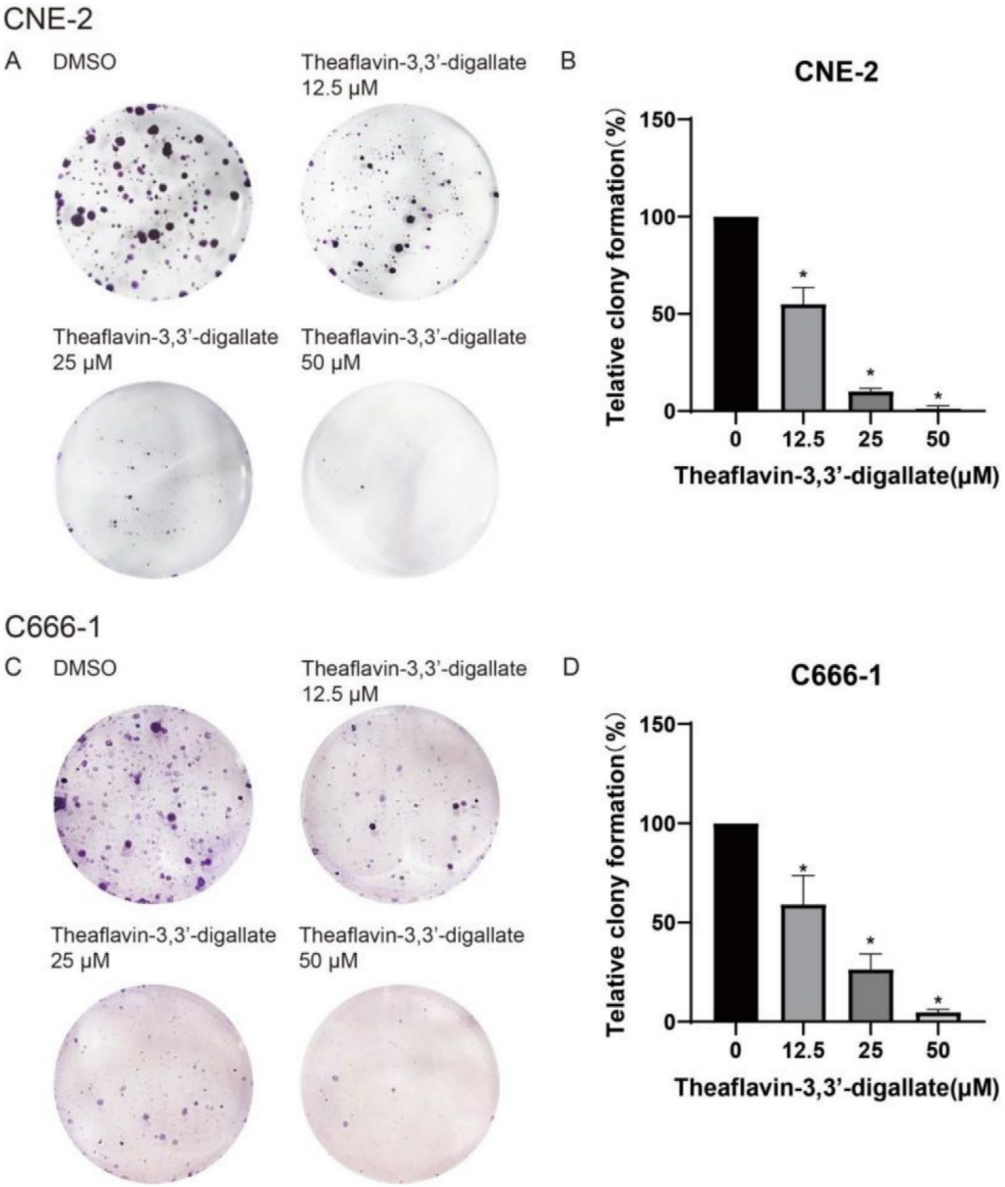
The effect of TF3 on the clonogenic ability of CNE‐2 and C666‐1 cells (*p < 0.05, *n*3).

TF3 may affect colony formation capability of CNE‐2 and C666‐1 cells was evaluated in this study. Consistently, when cells were treated with ascending concentrations ofTF3, the number of colonies was significantly declined at higher concentrations (50.0 μM) compared to the non‐treated cells (Figure 2).

### 3.3 TF3 Suppresses the Migration Ability of NPC CNE‐2 and C666‐1 Cells

**FIGURE 3:**
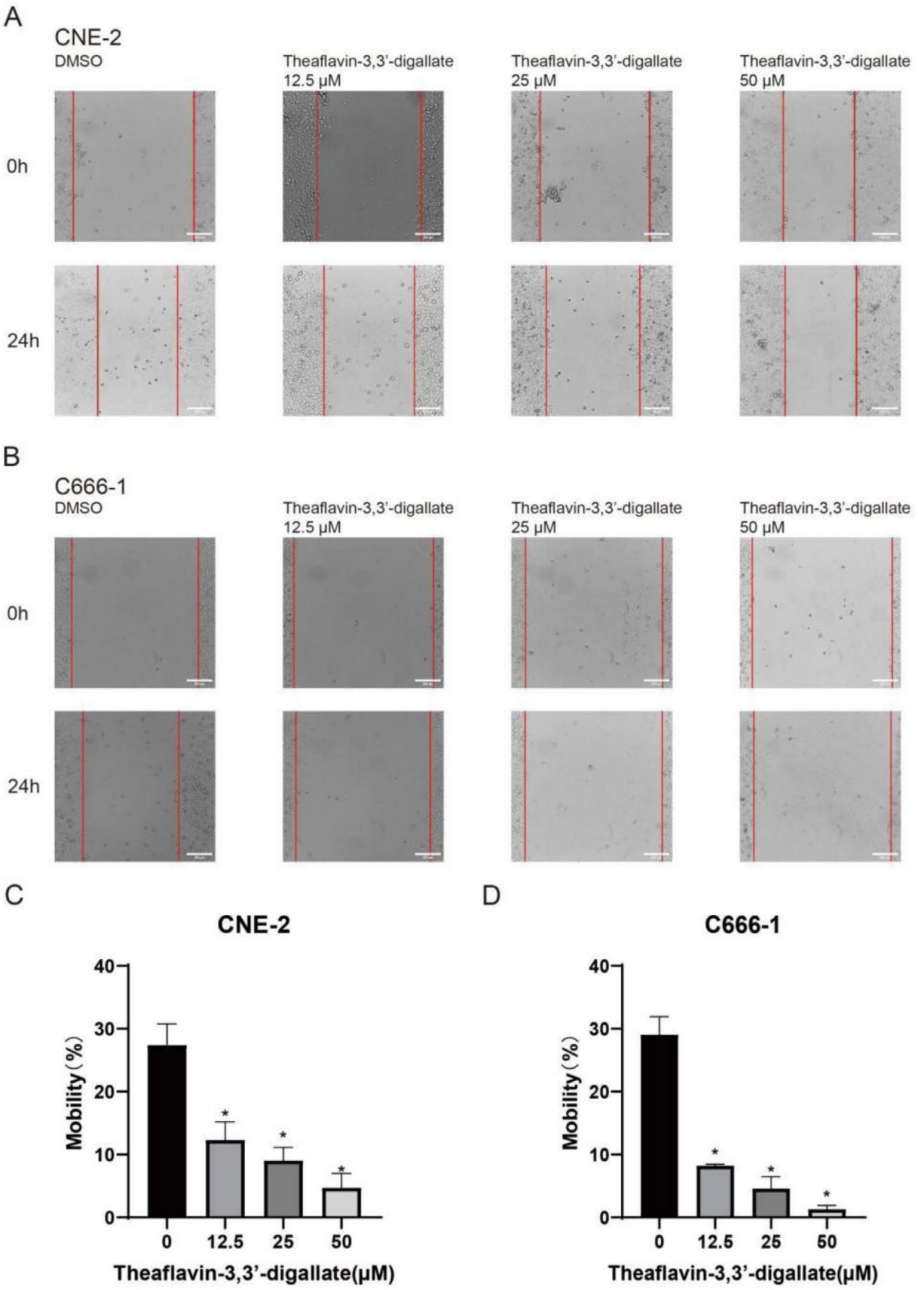
The effect of TF3 on CNE‐2 and C666‐1 cell migration (*p < 0.05, *n*3).

The effect of TF3 on the migration ability of CNE‐2 and C666‐1 cells was assessed using a scratch assay. Consistently, treatment with TF3 resulted in slower cell migration and exhibited a dose‐dependent relationship. The higher the TF3 concentration, the more pronounced the inhibition of cell migration. Significant results were observed at high concentrations (50 μM) (Figure 3).

### 3.4 TF3 Inhibits the Invasion Ability of NPC CNE‐2 and C666‐1 Cells

**FIGURE 4:**
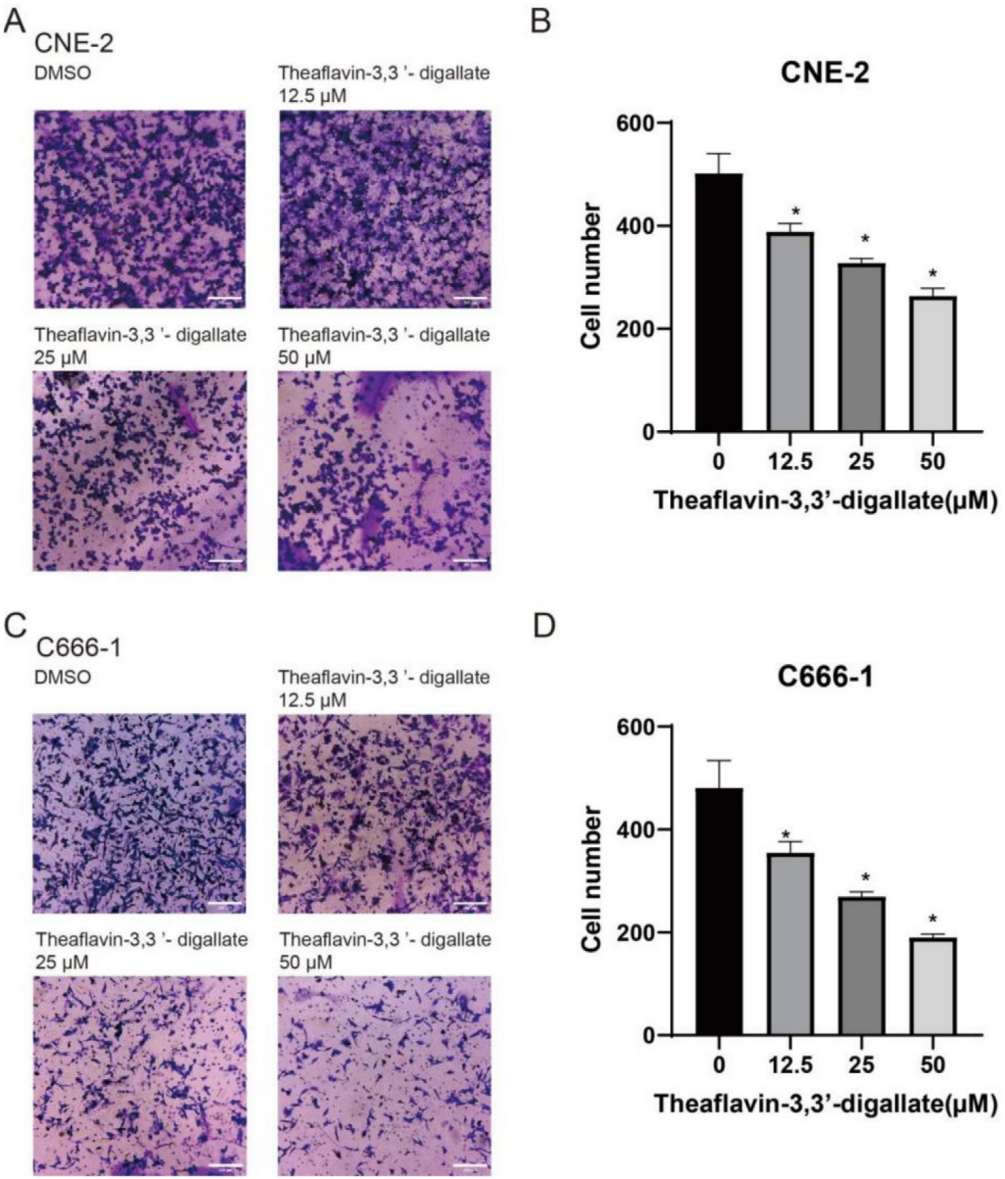
The effect of TF3 on cell invasion of CNE‐2 and C666‐1 cancer cell lines (*p < 0.05, *n*3).

The Transwell invasion assay was conducted to assess the quantity of cells traversing the polycarbonate membrane in response to varying medication concentrations. The findings indicated that an increase in drug concentration corresponded with a reduction in the number of cells traversing the polycarbonate membrane. TF3 demonstrated a more substantial suppression of invasion capability with escalating doses, with significant inhibitory effects noted at a medication concentration of 50 μM. The results demonstrate that TF3 exerts an inhibitory effect on the invasive capacity of NPC CNE‐2 and C666‐1 cells, exhibiting a dose‐dependent relationship (Figure 4).

### 3.5 TF3 Promotes Apoptosis in CNE‐2 and C666‐1 Cells in a Dose‐Dependent Manner

**FIGURE 5:**
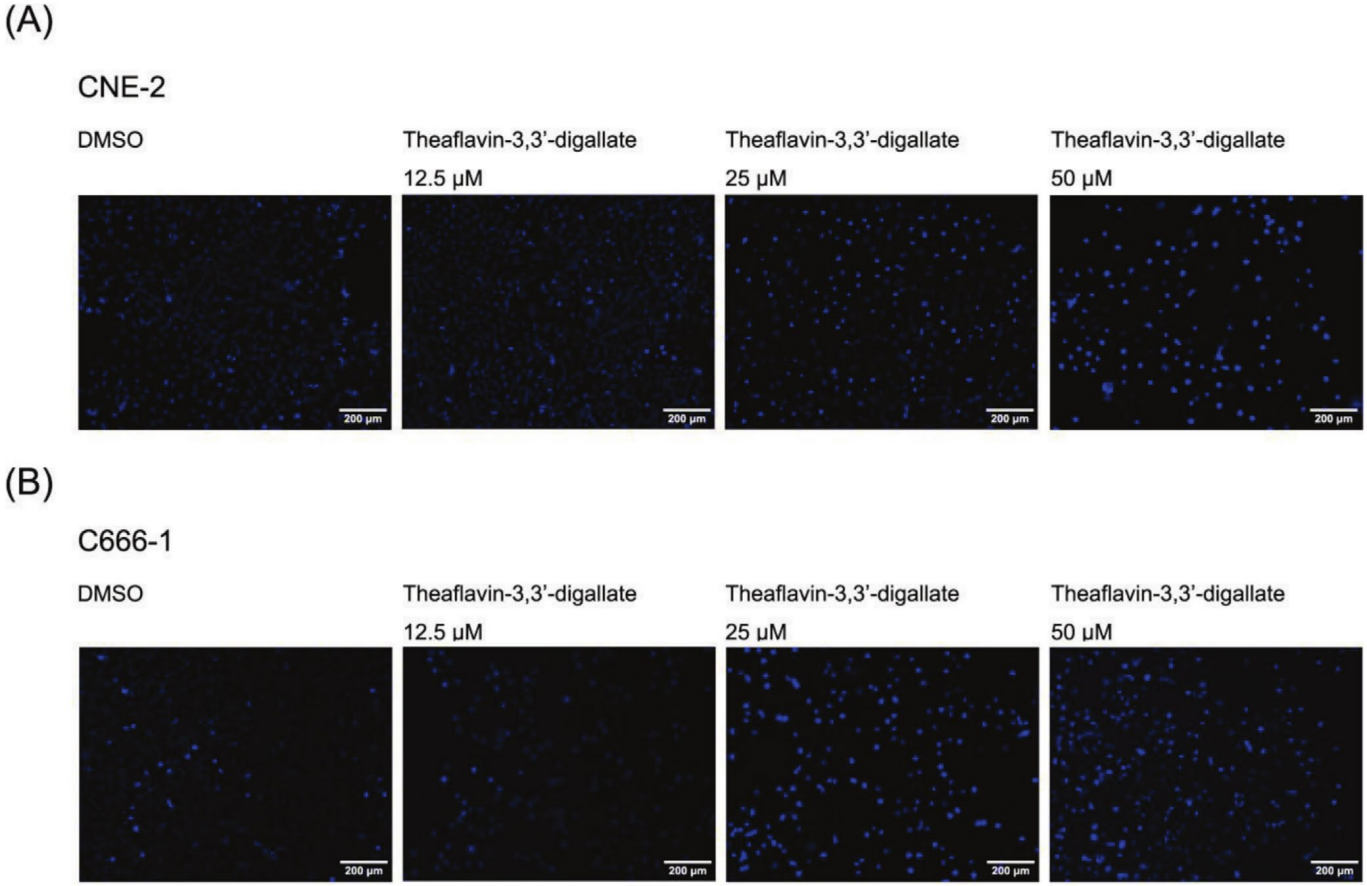
Morphological alterations in CNE‐2 and C666‐1 cells following TF3 exposure (*p < 0.05, *n*3).

CNE‐2 and C666‐1 cells, subjected to varying doses of TF3 for 24 h, were stained utilizing the apoptosis‐Hoechst staining kit. The findings indicated nuclear condensation and apoptotic characteristics, including cell shrinkage, in the TF3 treatment group of CNE‐2 and C666‐1 cells. Significant apoptotic effects were noted at a medication dose of 50 μM, suggesting that TF3 can induce apoptosis in CNE‐2 and C666‐1 cells (Figure 5).

### 3.6 Metabolite Identification Quantity Statistics and Inter‐Group Differential Analysis

**FIGURE 6:**
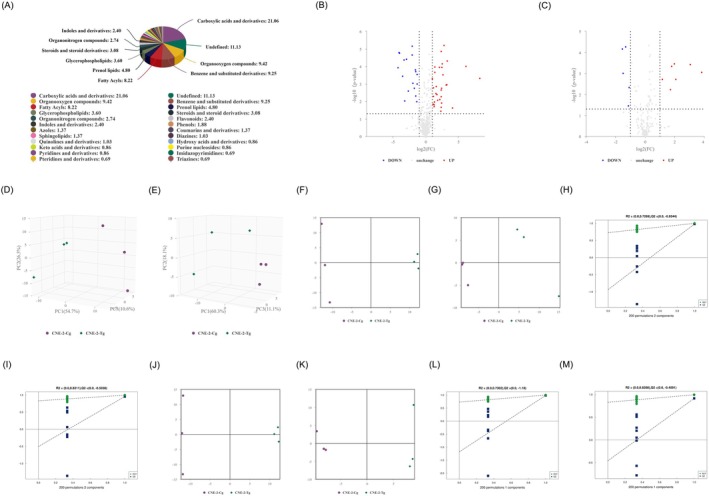
Metabolite identification quantity statistics and inter‐group differential analysis (CNE‐2‐Tg vs. CNE‐2‐Cg). (A) Proportional distribution of metabolites across different chemical classes. (B) Volcano plot displaying results for positive ion mode. (C) Volcano plot displaying results for negative ion mode. (D) PCA score plot for positive ion mode. (E) PCA score plot for negative ion mode. (F) PLS‐DA score plot for positive ion mode. (G) PLS‐DA score plot for negative ion mode. (H) PLS‐DA permutation test results for positive ion mode. (I) PLS‐DA permutation test results for negative ion mode. (J) OPLS‐DA score plot for negative ion mode. (K) OPLS‐DA score curve for negative ion mode. (L) OPLS‐DA permutation test results for positive ion mode. (M) OPLS‐DA permutation test results for negative ion mode.

About 584 metabolites were confirmed by integrating data from both positive (423 metabolites) and negative (161 metabolites) ion modes. All identified metabolites, combined from both ionization modes, were classified based on their Chemical Taxonomy. The distribution of different metabolite classes is presented in Figure 6A. Univariate analysis was performed on all detected metabolites, including those that were unidentified, in both positive and negative ion modes. Differential analysis was conducted for metabolites with a fold change (FC) > 1.5 or FC < 0.67 and a *p* value < 0.05. The results of this analysis were visualized using volcano plots, as shown in Figure 6B,C. To comprehensively capture the variation between sample groups and within groups, Principal Component Analysis (PCA) was employed. PCA analyses were performed for each comparison group, with an exemplar contrasting group illustrated in PCA score plots in Figure 6D,E. The PCA model parameters obtained through 7‐fold cross‐validation yielded R2X values of 0.918 in positive mode and 0.895 in negative mode, respectively. The discernible high intra‐group clustering and distinct inter‐group separation indicate a robust model with significant inter‐group differences. Partial Least Squares Discrimination Analysis (PLS‐DA) models were established to discriminate metabolites relevant to grouping. PLS‐DA score plots are presented in Figure 6F,G. The model evaluation parameters (R2Y, *Q*2) obtained through 7‐fold cross‐validation yielded *Q*2 values of 0.98 and 0.956, signifying model stability and reliability. To forestall overfitting during the modeling process, permutation tests were executed to ensure model validity. Figure 6H,I showcase the permutation test results for the PLS‐DA model of the comparison group, demonstrating a gradual decrease in *R*2 and *Q*2 with decreasing permutation retention, affirming the absence of overfitting and showcasing the model's robust performance. Further OPLS‐DA analysis was conducted (Figure 6J,K), and model evaluation parameters (*R*2Y, *Q*2) obtained through 7‐fold cross‐validation yielded *Q*2 values of 0.985 and 0.916, indicating a stable and reliable model. Permutation tests were employed to validate the OPLS‐DA model, as depicted in Figure 6L,M. The decreasing *R*2 and *Q*2 as permutation retention decreases confirm the absence of overfitting, attesting to the model's robustness.

### 3.7 Differential Metabolite Selection

**FIGURE 7:**
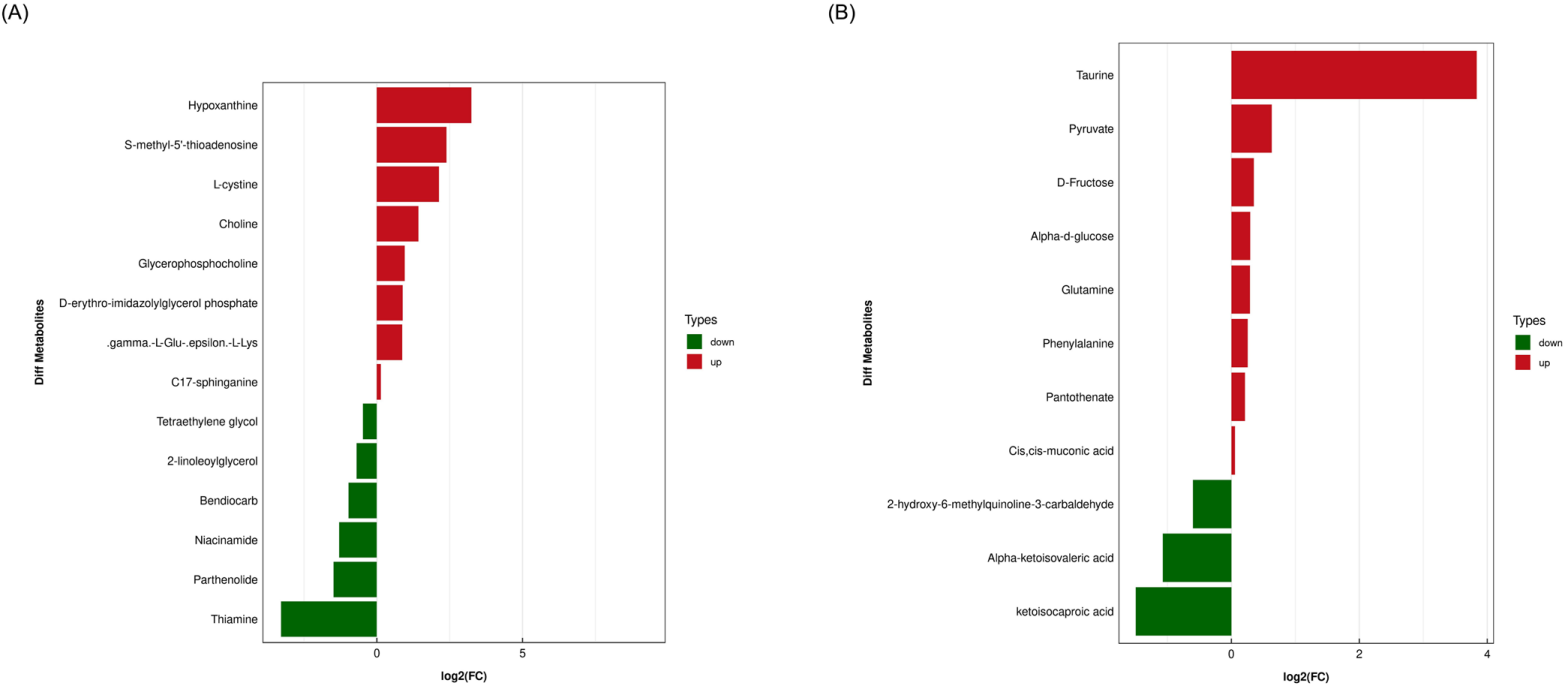
Differential metabolite selection (CNE‐2‐Tg vs. CNE‐2‐Cg). (A) Analysis of differential expression multiples for significant differential expression of metabolites in positive ion mode. (B) Analysis of differential expression multiples for significantly differentially expressed metabolites in negative ion mode.

Metabolomics studies typically employ stringent criteria such as OPLS‐DA VIP > 1 and *p* value < 0.05 for the selection of significantly differentially expressed metabolites. This experiment adheres to these criteria for screening purposes. The bar charts visually illustrate the fold change variations of identified significantly different metabolites, as shown in Figure 7A,B. In the positive ion mode, notable elevations were observed in the levels of metabolites such as Hypoxanthine, S‐methyl‐5′‐thioadenosine, L‐cystine, Choline, Glycerophosphocholine, D‐erythro‐imidazolylglycerol phosphate, gamma‐aminobutyric acid, and C17‐sphinganine. Conversely, Tetraethylene glycol, 2‐linoleoylglycerol, Bendiocarb, Niacinamide, Parthenolide, and Thiamine exhibited significant decreases in their levels. In the negative ion mode, noteworthy increases were detected in the levels of metabolites such as Taurine, Pyruvate, D‐Fructose, Alpha‐d‐glucose, Glutamine, Phenylalanine, and Pantothenate. Meanwhile, metabolites including 2‐hydroxy‐6‐methylquinoline‐3‐carbaldehyde, Alpha‐ketoisovaleric acid, and ketoisocaproic acid showed significant decreases in their levels.

### 3.8 Correlation Analysis of Differential Metabolites

**FIGURE 8:**
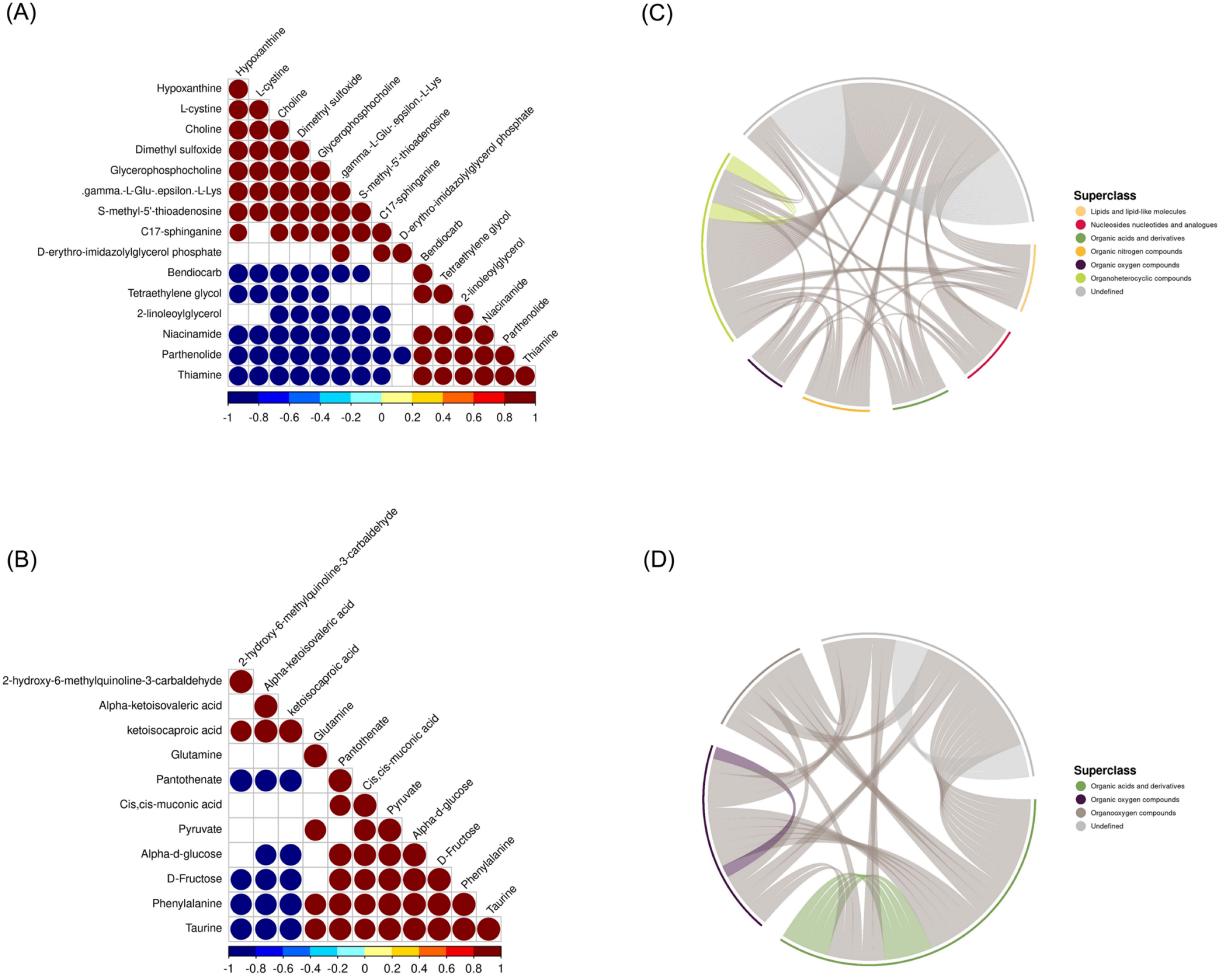
Correlation analysis of differential metabolites (CNE‐2‐Tg vs. CNE‐2‐Cg). (A) Correlation heatmap in positive ion mode. (B) Correlation heatmap in negative ion mode. (C) Chord diagram in positive ion mode. (D) Chord diagram in negative ion mode.

Correlation analysis plays a crucial role in gauging the metabolic proximity among significantly different metabolites (VIP > 1, *p* value < 0.05), providing valuable insights into the intricate regulatory relationships between metabolites during alterations in biological states. Metabolites exhibiting correlation may collaboratively partake in specific biological processes, indicative of functional relevance. Additionally, these metabolites may showcase cooperative or mutually exclusive relationships. For instance, a concordant trend in the changes of metabolites implies positive correlation, whereas opposing trends signify negative correlation. Synthetic transformation relationships are shown by negative correlations, which may indicate breakdown for the production of other metabolites, and positive correlations, which may indicate a shared biosynthetic pathway. We performed correlation analysis on the chosen metabolites that were significantly different, meeting the requirements of both OPLS‐DA VIP > 1 and *p* value < 0.05, in order to go on. Employing the Pearson correlation analysis method, we explored the interrelationships among these metabolites, visually presenting the results through correlation heatmaps and chord diagrams, as depicted in Figure 8A–D. In positive ion mode, diverse metabolites demonstrated robust correlations. Notably, there was a significant positive correlation between Hypoxanthine and L‐cystine, while Hypoxanthine exhibited a significant negative correlation with Thiamine. In negative ion mode, similar correlations emerged among significantly different metabolites. For instance, 2‐hydroxy‐6‐methylquinoline‐3‐carbaldehyde displayed a significant positive correlation with Alpha‐ketoisovaleric acid, while showing a significant negative correlation with Taurine.

### 3.9 Cluster Analysis of Differential Metabolites and KEGG Analysis

**FIGURE 9:**
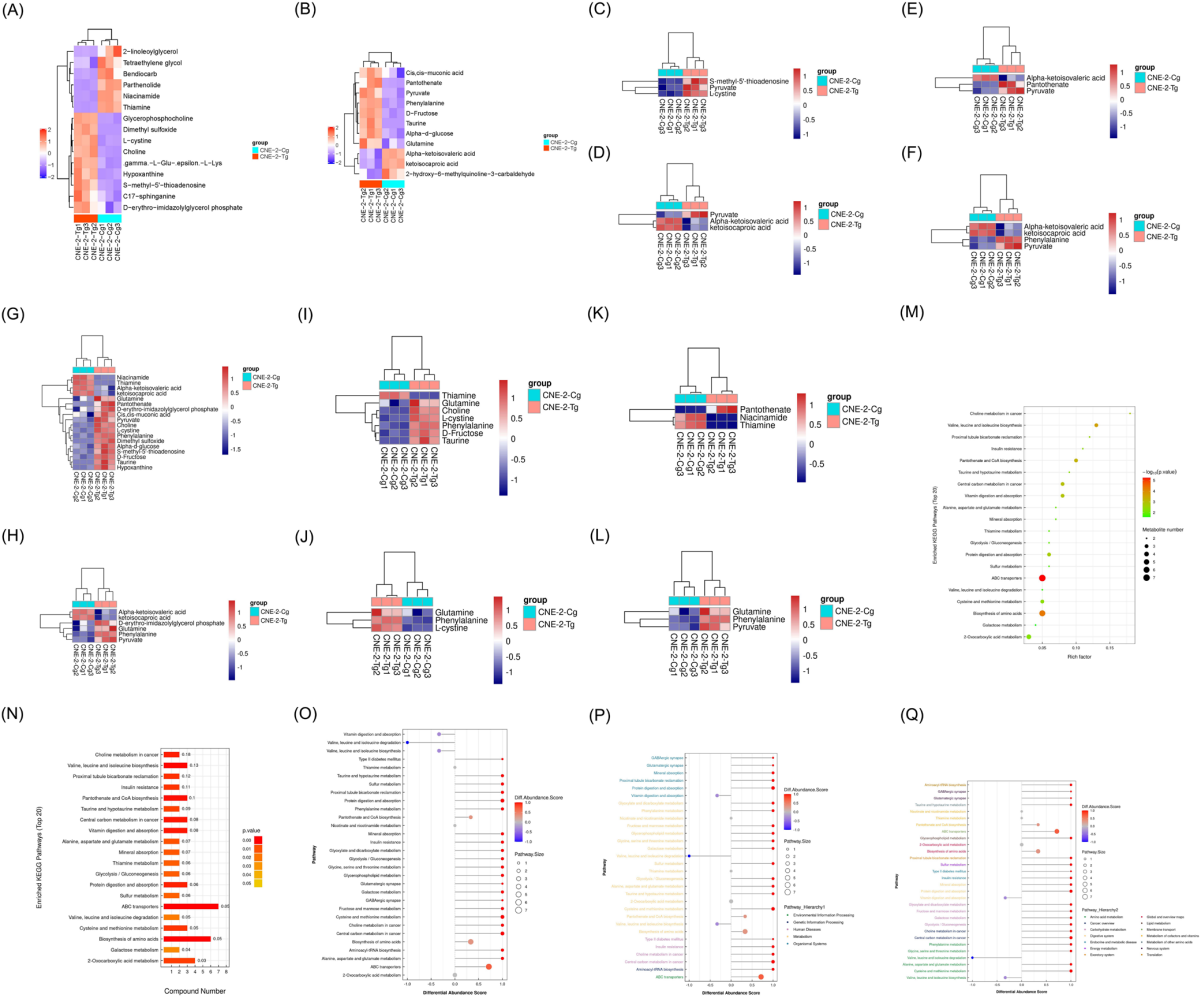
Cluster analysis of differential metabolites and KEGG analysis (CNE‐2‐Tg vs. CNE‐2‐Cg). (A) The heatmap of significantly different metabolites in positive ion mode hierarchical clustering. (B) A hierarchical clustering heatmap of metabolites exhibiting statistically significant differences in negative ion mode. (C–L) For KEGG metabolic pathways, the quantity of differential metabolites must exceed three. (M) The enhanced KEGG pathways are illustrated in a bubble chart. (N) A bar chart depicting KEGG pathway enrichment. (O) Graphs illustrating the differential abundance scores of each metabolic pathway. (P) Pathway hierarchy‐based differential abundance score maps for each distinct metabolic pathway. (Q) Graphs depicting differential abundance scores for each metabolic pathway classified by path hierarchy.

To accurately represent the relationships between samples and the differential expression patterns of metabolites, we standardized the expression levels of all samples and differential metabolites by dividing each group's root mean square by the average and subtracting the mean. Subsequently, we conducted hierarchical clustering analysis and calculated the distance matrix. The results of hierarchical clustering for metabolites that are clearly distinct from each other (VIP > 1, *p* value < 0.05) are illustrated in Figure 9A,B. In positive ion mode, relative to the normal control group, metabolites such as Tetraethylene glycol, 2‐linoleoylglycerol, Bendiocarb, and Niacinamide showed a significant decrease in tea polyphenol‐treated cells, while Glycerophosphocholine, Hypoxanthine, and gamma‐aminobutyric acid exhibited a significant increase. This suggests a close association of these metabolites with cell proliferation, migration, and invasion. To facilitate the observation of the expression patterns of differentially expressed metabolites annotated in KEGG metabolic pathways, according to Figure 9C–L, we chose to display heatmaps for KEGG pathways that had more than three divergent metabolites. Clusters of similarly expressed metabolites may represent functional homology or shared participation in a metabolic pathway or biological action. Figure 9M,N, respectively, show the findings of the metabolic pathway enrichment analysis in a bubble chart and a bar chart. The Differential Abundance Score captures the average overall fluctuation of all metabolites within a pathway and is a pathway‐based technique for assessing metabolic alterations. Figure 9O–Q displays the differential abundance scores for all metabolic pathways that were significantly enriched. The results reveal that the ABC transporter and biosynthesis amino acid pathways are significantly enriched, with the highest number of enriched metabolites. This suggests a crucial role for these two signaling pathways in the regulation of tea polyphenols in nasopharyngeal carcinoma cell proliferation, warranting further investigation.

## 4 Discussion

Malignant NPC tumor naturally arose in the epithelial cells of the nasopharyngeal mucosa (Saleh et al. 2021). It exhibits a highly malignant nature, characterized by early metastasis, posing a significant threat to human health. The incidence of NPC is relatively high in regions such as Guangdong, Guangxi, Fujian, and Hunan, particularly among the East Asian population. Due to the unsatisfactory outcomes of conventional chemotherapy and radiotherapy, it is imperative to explore alternative therapies that are less toxic and based on natural compounds.

New research suggests that theaflavins can promote apoptosis while inhibiting the growth of cancer cells, survival, and migration in vitro. Theaflavins induce cell death in NPCs, as shown by an upregulation of cleaved PARP (Alqahtani et al. 2019) and cleaved caspase‐3,‐7,‐8, and ‐9, according to a number of relevant research. To back up the anti‐proliferative actions of theaflavins, some studies have shown that after treatment with theaflavins, Bax expression is upregulated and Bcl‐2 expression is downregulated (Jing et al. 2022; Annadurai et al. 2013). These changes are linked to the suppression of NPC cells. In addition, the amounts of Akt phosphorylation (Kai et al. 2023), mTOR, and c‐Myc in cancer cells were reduced after theaflavin treatment (Dalei et al. 2022), which are typically overexpressed in normal cancer cells. Several studies have also shown that the tumor suppressor p53 (Motolani et al. 2023; Lianrong et al. 2023), which is often mutated or downregulated in cancer, is upregulated following theaflavin treatment. Moreover, theaflavins have been found to induce a decrease in migration and invasion, possibly due to a reduction in MMPs expression (Kong et al. 2015). Notably, some studies have suggested that the pro‐apoptotic effects of theaflavins are more pronounced in cancer cell systems compared to non‐cancer cell systems. This indicates that theaflavins may offer a more targeted approach to cancer treatment compared to conventional chemotherapy drugs.

In summary, numerous studies have demonstrated the anti‐cancer potential of tea extracts along with its key theaflavin isomers. Theaflavins exhibit significant anti‐proliferative, anti‐migratory, anti‐invasive, and pro‐apoptotic activities against various types of cancer in different tissues. Numerous in vitro studies have shown that theaflavins downregulate major signaling pathways associated with cancer characteristics while upregulating certain anti‐cancer factors. Moreover, several in vivo experiments have observed the anti‐cancer abilities of theaflavins in the tumor microenvironment, as evidenced by the reduction in tumor occurrence in various animal models. Interestingly, theaflavins are reported to pose multiple targeted effects in various tissues and cell types, with their anti‐proliferative and pro‐apoptotic abilities seemingly more pronounced in cancer cells in comparison to normal cells. Further research is warranted to explore different administration routes and drug formulations in order to maximize bioavailability and effectiveness. Additionally, the design of appropriate nutritional supplements could maximize the differential effects of theaflavins on cancer and non‐cancer cells, providing a novel approach for cancer prevention and treatment.

The comprehensive metabolomics profiling revealed significant metabolic alterations in tea polyphenol‐treated nasopharyngeal carcinoma cells, with 584 metabolites identified across both positive and negative ion modes. Differential analysis using stringent statistical criteria (VIP > 1, *p* < 0.05, FC > 1.5 or < 0.67) uncovered numerous up‐ and downregulated metabolites indicative of altered metabolic states. PCA, PLS‐DA, and OPLS‐DA models demonstrated strong intra‐group consistency and inter‐group separation, validated by robust *R* ^2^ and *Q* ^2^ values and permutation testing, confirming model stability and reliability. Significantly altered metabolites such as Hypoxanthine, L‐cystine, Glycerophosphocholine, Taurine, and Pyruvate suggest shifts in purine metabolism, amino acid metabolism, and membrane lipid turnover. Correlation and clustering analyses further supported the presence of co‐regulated metabolic networks, with KEGG enrichment pointing to the ABC transporter and amino acid biosynthesis pathways as key regulatory nodes influenced by tea polyphenol treatment. These findings underscore the potential mechanistic role of specific metabolic pathways in mediating the antiproliferative effects of tea polyphenols in nasopharyngeal carcinoma.

## 5 Conclusion

The present study was aimed to examine how TF3 affects human NPC CNE‐2 and C666‐1 cells in vitro, with a focus on their proliferation, migration, invasion, and the underlying mechanisms of its anticancer effects. Through CCK‐8 assays, colony formation assays, scratch assays, Transwell invasion assays, and Hoechst staining, it was confirmed that there were antiproliferative effects, reduced migration, and invasiveness against CNE‐2 and C666‐1 cell lines, along with a significant promotion in apoptosis rates. The effects were dose‐dependent, with increasing doses yielding more pronounced effects.

## Author Contributions

**Zihao Zhou:** methodology (equal), validation (equal), visualization (equal). **Chunpeng Wan:** investigation (equal), project administration (equal), writing – original draft (equal). **Yudi Gan:** validation (equal), visualization (equal), writing – original draft (equal), writing – review and editing (equal). **Xiaomeng Hu:** data curation (equal), formal analysis (equal), investigation (equal). **Puxiang Yang:** data curation (equal), investigation (equal), methodology (equal), resources (equal). **Zhonghua Liu:** investigation (equal), project administration (equal), resources (equal), supervision (equal), validation (equal). **Mingxi Li:** conceptualization (equal), resources (equal), writing – original draft (equal). **Muhammad Farrukh Nisar:** methodology (equal), validation (equal), writing – review and editing (equal). **Yi Cai:** project administration (equal), visualization (equal), writing – review and editing (equal).

## Conflicts of Interest

The authors declare no conflicts of interest.

## Data Availability

The data that support the findings of this study are available on request from the corresponding authors.
